# SARS-CoV-2 and regular patient treatment – from the use of rapid antigen testing up to treatment specific precaution measures

**DOI:** 10.1186/s13005-021-00289-9

**Published:** 2021-09-04

**Authors:** Jürgen Durner, Thomas Beikler, David C. Watts, Marc Becker, Miriam E. Draenert

**Affiliations:** 1 Department of Conservative Dentistry and Periodontology, University Hospital, LMU Munich, Ludwig-Maximilians-University Munich, Goethestr. 70, Goethestraße 70, 80336 Munich, Germany; 2 Laboratory Becker & Colleagues, Führichstr. 70, 81671 Munich, Germany; 3 Department of Periodontics, Preventive and Restorative Dentistry, University Medical Center Hamburg-Eppendorf, Martinistraße 52 (Building O58), 20246 Hamburg, Germany; 4 School of Medical Sciences and Photon Science Institute, University of Manchester, Manchester, UK

**Keywords:** COVID-19, SARS-CoV-2, Dental Treatment, Rapid Antigen Tests, Precaution, Dentistry, Dental Practice

## Abstract

**Introduction:**

The COVID-19 pandemic poses a continued challenge for all parties involved especially for the dentist as routine operation must be resumed. Rapid Antigen Tests (RATs) are actually recommended to identify and minimize infectious risks. However, there is still no guideline on the implementation of RATs in a dental or medical setting.

**Methods:**

Based on data and an extensive literature research regarding rapid antigen testing and reflecting the recommendations given by the various professional societies a task force was formed to determine a specific testing and treatment strategy.

**Results:**

A comprehensive test and treatment strategy and risk analysis was developed with practical suggestions for a wide range of typical activities in dental and medical offices.

The transmission of SARS-CoV-2 and its variants via aerosols and droplets as well as the difficulties to maintain the minimum distance form special challenges to the dental routine. RATs might in addition to optimal and necessary hygienic standards in combination with the use of adequate personal protection equipment be an important instrument in managing the challenges.

**Conclusions:**

The present work gives recommendations for dental routine operation (dental practices, outpatient clinics) to provide the necessary dental care for the population while protecting the doctor, practice team and patient at the same time.

## Introduction

The spread of severe acute respiratory syndrome coronavirus 2 (SARS-CoV-2) is classified as a pandemic by the world health organization (WHO). Recently, there have been several variants whose contagiosity seems to be increased compared to the wild type, such as B.1.1.7 (20I/501Y.V1) known as the UK variant or B.1.351 (20 H/501Y.V2) known as the South African variant. Close contact or short-range transmission of infectious saliva droplets is the primary mode for spreading SARS-CoV-2 [1]. Therefore, the dental team is at risk for infection since it is impossible to keep a minimum safe distance of 1.5 m during treatment or to use partition walls and the patients cannot wear face masks during treatment. However, this holds true for the patient as well, at least in part. Moreover, the use of water-cooled rotating, oscillating (sonic- or ultrasonic) and air-polishing devices can increase the risk for infection by generating pathogen-containing aerosols [2, 3]. The highest concentrations of contaminated aerosols are found located at the nose, the head, the chest, the arms and the mouthguard of dentists and dental assistants [4]. Interestingly, those aerosols can be spread over a range of 30 m in an indoor setting, e.g. a dental office, indicating potential long-distance saliva aerosol transmission [5]. Air circulation by ventilation and insufficient air conditioning systems may worsen the effect.

While vaccination against SARS-CoV-2 began in late 2020, the recent spread of more aggressive and potentially resistant variants may counteract vaccination effects and thus interfere with the reinstallation of a pre-pandemic dental routine [6].

Testing in this context is a practical tool for identifying potentially infected individuals. It allows not only to isolate and identify contact traces and thus reduce the spread, but also to select the appropriate measures concerning the handling of the respective individuals.

The analytical gold standard for the detection of the virus and some of the variants SARS-CoV-2 is the reverse transcription-quantitative polymerase chain reaction (RT-qPCR) from a nasopharyngeal swab or throat swab [7–9]. The RT-qPCR examination cannot, however, be carried out directly on the patient, since the sample must be transferred to the laboratory and the corresponding result of the analysis returned. The desire for a point of care (POC) testing system, independent of specialized staff, that allows on-site testing and a rapid result has led to the introduction of rapid antigen tests (RATs) [10]. However, the advantage of on-site testing via RATs is mitigated by lower sensitivity compared to RT-qPCR [7, 11]. Nevertheless, RATs can be administered by laypersons without specific training [12]. Thus, the use of RATs has been proposed in various contexts of daily life, such as in educational, working or recreational environments.

Surprisingly, no literature reference can be found on the implementation of RATs within the testing and treatment strategy of a dental practice. Therefore, the aim of this study was to develop a test and treatment strategy that enables a dental practice to function during periods of SARS-CoV-2 prevalence by safeguarding to a maximum safety level for both patients and the dental care team.

## Methods

Following an extensive literature research, regarding rapid antigen testing and reflecting the recommendations given by the various professional societies, a joint group was formed to establish a specific testing and treatment strategy. The key word searches in PubMed and the Cochrane Library, for articles published up to March 2021, were combinations of: dentistry, dental surgery, dentist, dental, patient, corona, SARS-CcV-2, rapid antigen test, dental personal, influence test strategy.

## Results

A comprehensive test strategy must be applicable to both patients and all dental staff.

### Dental Personal

The dentist and his/her staff should undergo a basic triage.

1. For symptom-free dentists and dental staff a RAT is recommended, with a regularity dependent on the prevalence.
2. In suspicious cases RATs can be applied to identify a potential infection.
3. Should the dentist or his/her staff show any signs of typical symptoms such as fewer, cough, sore throat, hoarseness, diarrhea or have been in contact with a SARS-CoV-2 positive persons within the last two weeks they should stay at home and should be tested by RT-qPCR.
4. Should, after treatment, it be determined that a patient has a positive test result regarding SARS-CoV-2, all contact persons should be tested, dependent on the duration, type of treatment and protective measures in place. The steps to be taken should be in line with the requirements of the local authorities.

Asymptomatic carriers are of particular importance, as they can pass on the virus without becoming ill themselves and can contribute to the transmission of the virus [13]. This is particularly important because there are immunosuppressed patients, e.g. after organ transplantation, who cannot build up antibody protection despite vaccination [14]. To minimize the respective risk, it is recommended to test the dentist and his staff on a regular and frequent base. The frequency with which RAT are performed should be adjusted (twice or more a week) depending on the actual infection level, prevalence and be consistent with guidelines of the respective authorities.

### Patients

It is recommended that all patients are triaged before any appointment (preferably by phone and prior to entering the dental office) to determine their SARS-CoV-2 status, i.e. whether they show any typical symptoms such as fever, cough, sore throat, hoarseness, diarrhea or have been in contact with SARS-CoV-2 positive persons within the last two weeks (suspected of SARS-CoV-2) or have been tested positive for SARS-CoV-2 [2].

Based on the information received the following patient groups can be identified:

A) patients without any signs of SARS-CoV-2 infection.
B) patients suspected of SARS-CoV-2 infection regardless of whether they have been already vaccinated or had COVID-19 previously.
C) patients tested positive for SARS-CoV-2.

When entering the dental office all patients of group A) should be invited to undergo a RAT. If such a patient refuses to take the test, a healthy carrier status cannot be excluded.

A RAT is strongly advised for patients of group B). If such a patient refuses to take a RAT, the patient should be regarded as if they tested positive.

For patients in groups A and B who refuse a RAT, the treatment-option benefits of a test should be explained.

Patients of group C) should not be re-tested.

Whenever the RAT is positive the patient should be isolated immediately, the necessary authorities contacted and an RT-qPCR-test completed. Only emergency treatments should be performed.

This general procedure is straightforward and flexible to apply and should maximize safety.

### Treatment strategy

The treatment strategy should allow for the maximum of dental treatment possibilities given the respective risk assessment status of a SARS-CoV-2 infection.

The following considerations should be taken into account and adapted if necessary.

For patients of group A) who have a negative RAT and are without any signs of SARS-CoV-2 infection all dental treatments can be conducted under the usual safety for all parties involved.

For patients of the group B) with a negative RAT and patients of group A) who refuse to have a RAT any elective treatment should be minimized. Dental treatment should be limited to dental therapies that have been started, and need to be finished, and to emergencies only. The reasoning for this is to minimize the contact time and reduce aerosol producing treatments as far as possible. The restriction of treatment for patients in group B is also based on the fact that they should not be subjected to additional burdens due to existing signs of the disease and that RATs can be false negative, especially at a low virus load particularly shortly after infection [15].

In patients of group C) and for those patients of group B) who refuse to be tested or have a positive RAT, the treatments are limited to dental emergencies only. This follows the strategy of minimizing the contact time as well as the duration of aerosol producing treatments [16]. The patients receiving treatment would be cases under severe pain, having a bacterial infection or suffering from consequences of an accident.

For all patients it is recommended that apart from hand washing and disinfection they wear Mouth Nose protective mask, of at least (FFP)2 quality, with mask removal only during actual intra-oral treatment.

For the dentist and the dental assistant in addition to intensive hand washing and disinfection (each 30 s), use of disposable gloves and multiuse gowns, the following additional personal protection equipment (PPE) and procedures are recommended to be used for all patient groups:

- FFP2 mask.
- safety goggles / visor or face shields.
- antiseptic mouth rinse prior to treatment for 30–60 s with for example 1 % H_2_O_2_ [3] or Listerine® cool mint [17].
- use of rubber dam whenever possible.
- avoidance of using aerosol generating devices when possible,
- preference for hand instrumentation.
- intensive surface cleaning and disinfection

In addition to the above precautions, it is advised to use additional PPE (FFP2/ mask, single use gowns and bonnets etc.) with patient groups B and C [16]. Dental care providers belonging to any risk group should critically calculate the risk and balance reasons whether to treat or not to treat SARS-CoV-2 positive or suspect patients.

The focus in Table 1 is on possible measures during treatment. Furthermore, it should be considered whether protective measures for front desk staff should be taken at the entrance of the practice. It should also be considered that as few patients as possible should be in the waiting area. The time interval between two treatments in a room should be chosen sufficiently to allow disinfection measures to be carried out and to adequately ventilate. Appointments and updates should be made using digital communication [16].

**Table 1:** Possibilities for clinical routine during a SARS-CoV-2 pandemic to protect patients and the dental team. It is particularly important to identify potential risks and to identify measures to minimize risks that an asymptomatic carrier infects another person directly or indirectly

No	Group/Location	Discipline	Activities	Risk	1. Substitution	2. Technical measures	3. Organizational measures	4. Personal protective equipment (PPE)
Front desk								
1)	Receptionist/Lobby	Appointments, conversations		Infection risk by transmission via droplets if the minimum distance cannot be maintained.	Clarify as much as possible by phone in advance	Protective screen		Mouth nose protective mask/FFP2 mask
2)	Receptionist/Lobby	Receipt of patient data via Chipcard		Contact transmission	Not possible	Card reader in front of screen. Card handled by patient.	No handing out of chip card, No physical contact, in case of contact hand disinfection.	Mouth Nose protective mask/FFP2 mask
Preparation work								
3)	Dental Assistant/ Waiting room/ hallway	Patients are called in and guided to the Treatment room		Infection risk by transmission via droplets if the minimum distance is not maintained.	Not possible	None	Maintain minimum distance of 1.5 m. No physical contact	Mouth Nose protective mask/FFP2 mask
4)	Dental Assistant/ Treatment room	Preparation work such as put on a napkin, patient cape		Infection risk by transmission via droplets as the minimum distance cannot be maintained.	Not possible	None	1% H_2_0_2_ Mouth wash for 60 s before treatment by patient	Mouth Nose protective mask/FFP2 mask
Diagnostic & Non-invasive Activities								
5)	Dentist/Treatment room	Activities, without invasive treatment, such as diagnosis consultation		Infection risk by transmission via droplets as the minimum distance cannot be maintained [3, 4]	Not possible	None	1% H_2_0_2_ Mouth wash for 60 s before treatment by patient	Mouth Nose protective mask/FFP2 mask, Eye protection (Goggle, Visor), Gloves. Caps, Single use cape [3, 18]
6)	Dental Assistant/Dentist/Treatment room	Radiograph		Infection risk by transmission via droplets as the minimum distance cannot be maintained.	Not possible	In patients with more gagging avoid optional intraoral x-ray, If necessary make an orthopantomo-gram	1% H_2_0_2_ Mouth wash for 60 s before treatment by patient	Mouth Nose protective mask/FFP2 mask
7)	Dental assistant/ Dentist/ Treatment room	Handling of the Dental impression		Contact transmission	Not possible Optional digital dental impressions	Rinse with water and disinfection solution	-	Mouth Nose protective mask/FFP2 mask, Eye protection (Goggle, Visor), Gloves. Caps, Single use cape [3, 18]
Invasive Activities								
8)	Dental Hygienist	Prophylaxis(tooth cleaning) Scaling and root planning, Polishing, working with rotating instruments, sonic-/ ultrasonic, air abrasion tool		Infection risk by transmission via droplets and aerosols as the minimum distance cannot be maintained [3, 4]	Possible	Reduction of aerosol development using only manual instruments(non sonic/ ultrasonic)	Postpone to a later date,	Mouth Nose protective mask/FFP2 mask, Eye protection (Goggle, Visor), Gloves. Caps, Single use cape [3, 18]
9)	Dental Assistant/Dentist/ Treatment room	Periodontal initial treatments/ recalls	Scaling and root planning, working with rotating instrument, sonic-/ ultrasonic, air abrasion tool	Infection risk by transmission via droplets and aerosols as the minimum distance cannot be maintained [3, 4]	Not possible	Reduction of Aerosol development using predominantly manual instruments	1% H_2_0_2_ Mouth wash for 60 s before treatment by patient	Mouth Nose protective mask/FFP2 mask,Eye protection (Goggle, Visor), Gloves. Caps, Single use cape [3, 18]
10)	Dental Assistant/ Dentist/ Treatment room	Restorative Treatment:Direct/ Indirect restaurations	working with rotating and sonic-/ ultrasonic equipment	Infection risk by transmission via droplets and aerosols, as the minimum distance cannot be maintained [3, 4]	Possible	Mouth covered using the dental dam. Reduction of aerosol development minimal use of sonic and ultrasonic instruments and turbine driven instruments.	1% H_2_0_2_ Mouth wash for 60 s before treatment by patient	Mouth Nose protective mask/FFP2 mask,Eye protection (Goggle, Visor), Gloves. Caps, Single use cape [3, 18]
11)	Dental Assistant/Dentist/ Treatment room	Prothetic Dentistry:	working with rotating and sonic-/ ultrasonic equipment	Infection risk by transmission via droplets and aerosols, as the minimum distance cannot be maintained [3, 4]	Not possible	None	1% H_2_0_2_ Mouth wash for 60 s before treatment by patient	Mouth Nose protective mask/FFP2 mask,Eye protection (Goggle, Visor), Gloves. Caps, Single use cape[3, 18] Optional prior Covid test recommended for the patient in case of larger preparations (several teeth)
12)	Dental Assistant/Dentist/ Treatment room	Endodontic Treatment	working with rotating and sonic-/ ultrasonic equipment	Infection risk by transmission via droplets and aerosols, as the minimum distance cannot be maintained [3, 4]	Not possible	Mouth covered using the dental dam. Desinfection of the tooth prior trepanation. Reduction of aerosol development minimal use of sonic and ultrasonic instruments and turbine driven instruments.	1% H_2_0_2_ Mouth wash for 60 s before treatment by patient	Mouth Nose protective mask/FFP2 mask,Eye protection (Goggle, Visor), Gloves. Caps, Single use cape[3, 18]
13)	Dental Assistant/Dentist/ Treatment room	Orthodontics	Brackets insertion or removal	Infection risk by transmission via droplets and aerosols, as the minimum distance cannot be maintained [3, 4]	Not possible	None	1% H_2_0_2_ Mouth wash for 60 s before treatment by patient	Mouth Nose protective mask/FFP2 mask,Eye protection (Goggle, Visor), Gloves.Caps, Single use cape[3, 18]
14)	Dental Assistant/Dentist/ Treatment room	Oral Maxillo Facial surgery	working with cutting/ oscillating/ rotating and sonic-/ ultrasonic equipment	Infection risk by transmission via droplets and aerosols, as the minimum distance cannot be maintained [3, 4]	Not possible	Postpone elective therapies in periods with high infection rates	1% H_2_0_2_ Mouth wash for 60 s before treatment by patient	Mouth Nose protective mask/FFP2 mask,Eye protection (Goggle, Visor), Gloves.Caps, Single use cape [3, 18]Optional prior Covid test recommended for the patient in case of elective surgery with increased aerosol developments

## Discussion

With the lifting of restrictions, normal life is returning and elective dental treatments with the above outlined risks need to be completed [19]. However, there are potentially new mutants that are classified by the WHO as variants of interest (VOI) or variants of concern (VOC), such as the lambda variant emerging in South America [20. Apart from new variants that can penetrate better or evade the immune response more easily, prevention is also useful, as little is known about the consequences of COVID-19 infection, currently known as long-COVID.

On the other hand, various questions need to be answered:

### What will be the future scenario?

At this point it is not clear how the lifting of restrictions will affect the spreading of SARS-CoV-2. There are many open questions such as:

a) Since studies show that the anti-body level reduces over a period of time, is immunity established for vaccinated persons or persons after passing COVID-19 and how long will it prevail [21]? Are all vaccines equally effective [22]? How often do new variants require revaccination? Can memory cells be formed that trigger a humoral immune response even after a long period of illness [21]?

From the dentist perspective: Can I treat SARS-CoV-2 harboring persons without getting infected or spreading the disease myself?

From the patient perspective: Can I get SARS-CoV-2 from the dentist/dental team?

b) How to address the risk regarding the spread via non-symptomatic carriers? Studies show that especially children and young adults fall frequently in this group and help spread the virus without having any signs of the disease.

The spread of non-symptomatic carriers can be reduced by adhering to strict hygienic measures and by frequent tests. Symptomless spreaders can also be vaccinated persons. Vaccinated persons have a lower viral load, but the extent to which they can transmit the disease is unclear and therefore not excluded [23].

c) Will a seasonal resurgence of the virus occur causing the same problems as seen today? What effect will current variants from the UK, South Africa or Brazil and potential future variants have? Will the immunity level established be effective also against those or future variants [24]?

Because of the unanswered questions, mentioned above, a lot of uncertainties will continue to exist in the dental world. However, patients and dental providers will continue to be at risk of infection but patients need to be treated and therefore prevention of infection will continue to be a high priority in the future.

### How to address these risks?

#### Identification of high-risk groups

It is essential to protect those who are at high risk of suffering from serious sequalae from COVID-19. The definition of this risk group is constantly being expanded. Initially, people older than 60 y or with cardiological or respiratory diseases were assumed to be at risk, but now hypertension, renal diseases, overweight people and smokers are also included. Irrespective of the ongoing definition, these patients again should critically balance the reasons whether elective dental treatment can be postponed. But the severe course of the disease may also be found in patients with no underlying preconditions and may also occur in young patients [25]. In addition, there are further groups of patients at higher risk like hemodialysis patients [26] or the group of immunosuppressed patients such as organ transplant patients or patient under immunosuppressive therapy for liver and intestinal diseases [27]. These are at risk in several respects. They have more frequent contact with doctors (practice, hospital) due to their illness, but their immune systems are no longer as efficient due to immunosuppression.

#### Identification of carriers

Any patient who is in general good health might also be an asymptomatic carrier of SARS-CoV-2, especially as the viral load is the highest in the early stages [2, 28]. This poses an increased threat for all persons. Therefore, it is necessary to test as many and as frequently as possible and ideally before every treatment. In this context, it is also helpful to take a travel history and ask about infections in their immediate environment (friends, work colleagues…).

While in other contexts of daily life - such as airlines, schools, restaurants and concerts - access is only granted for those who have tested negative, the question arises, why this should not also hold true for elective dental treatment [29]? The proposed test and treatment strategy should incentivize patients, enabling a wider range of dental procedures and safeguarding emergency treatment.

While frequent testing is helpful to navigate through these times, in most countries from a legal perspective nobody - neither dentist, employees nor patients - can be forced to be tested.

The dentist is however free to offer treatment to the patient and to manage his/her staff based on the respective risk evaluation. This could also minimize potential liability risk to the dentist from patients or employees. Any test can give false positive and false negative results so the clinical assessment of COVID-19 prevalence is also important. Also, due to the reduced sensitivity of RAT compared to RT-qPCR, the test frequency is important [30, 31]. The limitations can be overcome by increasing the test frequency [32].

To set up a RAT infrastructure within the dental practice and to perform those tests has associated costs. The dentist should be well equipped to incorporate a RAT in the practice organization. A RAT is in principle a kind of a PPE and the costs could be managed along with extra costs associated with SARS-CoV-2.

### Limitation of predictive value of RAT

According to the WHO and *European Centre for Disease Prevention and Control* (ECDC), RATs have a minimum performance requirement set at ≥ 80 % sensitivity and ≥ 97 % specificity, which is lower than those for RT-qPCR tests [33]. They perform best in cases with high viral load, in pre-symptomatic and early symptomatic cases. This means that in the very early stages of the infection they don’t perform as well. The results of the RAT have always to be seen in conjunction with the actual prevalence settings. In high prevalence settings RAT will have a high positive predictive value (PPV) and low negative predictive values NPV [34]. If you take for example a test with a sensitivity of 80 % and a specificity of 98 % and a prevalence of 20 % the PPV is 0.909 and the NPV is 0.951 or in other words 9 out of 100 have a false positive result and 5 out of 100 have a false negative result. While at 10 % prevalence the values are PPV = 0.816 and NPV = 0.978 respectively. Here 18 out of 100 might have a false positive result and 2 out of 100 a false negative result. At 1 % prevalence the rates for PPV are 0.288 and for NPV are 0.998. In 71 out of 100 cases the positive result is false and 2 out of 1000 cases show a false negative result. This means that in settings of high prevalence a positive test result will be very likely be true. In low prevalence settings a negative test is likely to be true, whereas a positive RAT should, due to the higher accuracy of RT-qPCR tests, always be confirmed with this test system. In general RATs can help to reduce transmission through early detection of highly infectious cases and enable a rapid start of contact tracing.

It is also unclear whether vaccinated persons or persons who have been infected are “safe” [35]. Recent studies show that the antibody level diminishes over time, so that it remains unclear how long this protection might actually last. Cases are described in the literature where even vaccinated persons can become re-infected [36].

Tracing apps can help identify people being exposed to an infected person [37]. But their effectiveness remains to be proven. Therefore, practical and workable measures must be developed and implemented so RATs may be a helpful adjunct to establish a safer daily routine. The advantage of RATs, apart from low cost, is their availability and flexibility, requiring no infrastructure, their ease of handling and rapid results.

The limitation of this study is that the test and treatment strategy for the operation of a dental practice during SARS-CoV-2 prevalence is based on the effectiveness of RAT to detect the prevailing variants. Another limitation is that there are no decision-making guidelines, as for other infectious diseases, that can be adapted for (dental) practices within the framework of the work.

To incorporate RAT in the dental practice, aspects like additional time requirements, the incorporation of the test results in the practice workflow, additional and trained staff, space requirements need to be addressed as well as the question about the associated additional costs.

## Conclusions

The fairly low incidence of infected dentists may indicate that dentists who are used to stringent hygienic standards and use of them consistently are not at higher risk. However, further studies need to confirm that observation. Given the wide scope of dentistry, those studies should not only include the number of dentists tested positive compared to other medical groups, but also consider the specific variables of the various disciplines (dental prosthetics, orthodontics, oral-maxillofacial surgery, periodontology, endodontics, restorative dentistry etc.) with different aerosol producing activities or possibility using rubber dam. Until those results are available, it appears to be imperative to maintain or further improve on those standards [3].

As the virus will be present in the foreseeable future it is necessary to establish safe routines for the dentist, his/her staff, and the patients to identify the respective carriers and to offer treatments based on the specific risk assessment. RATs, despite their limitations, could help the dentist manage the situation.

Finally, these measures are an additional cost to the practice if not reimbursed. Nevertheless, no savings should be made on security.

## Data Availability

All data and their sources are clearly shown.

## Abbreviations

COVID-19: Corona Virus Disease 2019
ECDC: European Centre for Disease Prevention and Control
FFP: Filtering Face Piece
NPV: Negative Predictive Value
POC: Point of Care
PPE: Personal Protection Equipment
PPV: Positive Predictive Value
RAT: Rapid Antigen Test
RT-qPCR: Reverse Transcription-quantitative polymerase chain reaction
s: Second
SARS-CoV-2: Severe acute respiratory syndrome coronavirus 2
WHO: World Health Organization
y: Year
